# Editorial: Circadian Rhythms and Metabolism

**DOI:** 10.3389/fendo.2017.00201

**Published:** 2017-08-15

**Authors:** Etienne Challet, Andries Kalsbeek

**Affiliations:** ^1^Clocks Team, Institute of Cellular and Integrative Neurosciences, UPR3212, Center National de la Recherche Scientifique (CNRS), University of Strasbourg, Strasbourg, France; ^2^Department of Endocrinology and Metabolism, Academic Medical Center (AMC), University of Amsterdam, Amsterdam, Netherlands; ^3^Hypothalamic Integration Mechanisms, Netherlands Institute for Neuroscience (NIN), Amsterdam, Netherlands

**Keywords:** circadian clock, clock gene, feeding, mitochondria, exercise, circadian desynchronization, cancer, Alzheimer’s disease

**Editorial on the Research Topic**

**Circadian Rhythms and Metabolism**

One of the major breakthroughs of the last decade in the understanding of energy homeostasis is the identification of a reciprocal control between circadian rhythmicity and cellular metabolism. Circadian rhythmicity is a fundamental endogenous process of almost every organism living on Earth. For instance, the alternation of hunger and satiety is not continuous over 24 h, but is instead structured in time along the light/dark cycle. In mammals, the temporal organization of metabolism, physiology, and behavior around 24 h is controlled by a network of multiple cellular clocks, synchronized *via* neuronal and hormonal signals by a master clock located in the suprachiasmatic nuclei of the hypothalamus. This central circadian conductor in the brain is mainly reset by ambient light perceived by the retina, while secondary circadian clocks in other brain areas and peripheral organs can be reset by meal timing. Chronic disruption of circadian rhythms, as seen in human shift-workers (up to 20% of the active population), has been associated with the development of a number of adverse mental and metabolic conditions. Understanding of the functional links between circadian desynchronization and overall health in animal models and humans, however, is still scarce. Interactions between circadian clocks and metabolism can occur at different levels: the molecular clockwork, internal synchronization *via* neurohormonal signals, or external synchronization *via* photic or feeding cues. This Research Topic comprises a number of reviews as well as research and methods articles that feature recent advancements in the mechanisms linking circadian clocks with energy metabolism, and the pathophysiological implications of these interactions for metabolic health.

The first chapter by Atger et al. is a thorough review on the relative contribution of circadian clocks and feeding cues on rhythmic transcriptional, translational, and posttranslational processes. In addition, they present the recently identified circadian interactions between gut microbiota and their hosts.

The next chapter by Dyar and Eckel-Mahan highlights the emerging contribution of metabolomics to the field of chronobiology to understand the mechanisms underlying circadian physiology and to develop tools for personalized medicine, such as new biomarkers and assessment of circadian phase.

Manella and Asher summarize recent advances in the circadian regulation of the mitochondria. Actually, most aspects of mitochondrial function and even morphology display circadian variations. The possibility of an autonomous mitochondrial oscillator is also discussed.

Then, Tsang et al. critically reviewed the advantages and limitations of various transgenic strategies to study the impact of disrupting circadian rhythms on energy metabolism.

Following these overviews, the next articles will be presented according to the organ and rhythmic function they refer to. While the suprachiasmatic nuclei of the hypothalamus are unanimously recognized as the site of the master clock, the role of the secondary clocks in the brain remains largely elusive. Mieda et al. assessed the contribution of the ventral forebrain in circadian rhythms. More specifically, they discovered that the daily patterns of locomotor activity, sleep, and feeding are disrupted in mice with a targeted deletion of the core clock gene *Bmal1* in the ventral forebrain, leaving the master clock in the suprachiasmatic nuclei functional. These findings reveal that the circadian clocks in the ventral forebrain participate in the circadian control of behavior and physiology.

Food intake is regulated by a balance between orexigenic factors, stimulating the sensation of hunger and foraging, and appetite-suppressing factors which, in contrast, favor satiety. Among the orexigenic molecules, there are circulating factors such as ghrelin released from the stomach, as well as several neuropeptides, such as orexin, neuropeptide Y, agouti-related peptide, melanin-concentrating hormone, and relaxin-3. In their review, Blasiak et al. highlight the cross talk between circadian rhythms and these orexigenic neuropeptides, and their interactions with the hypothalamic–pituitary–adrenal axis. They provide a focused review on the functioning of the aforementioned orexigenic neuropeptide systems and how these neurochemical pathways are affected by chronic stress and chronodisruption, leading to eating disorders and metabolic disturbances.

Ingestion of food triggers secretion of several hormones, like insulin from pancreatic β cells and leptin from the adipose tissue, both driving a loss of appetite by their activation of neuronal pathways releasing anorexigenic neuropeptides, such as melanocortin. Butler et al. provide a comprehensive review of the central melanocortinergic system with an emphasis on the role of the melaconocortin-3 receptor in the regulation of energy homeostasis and the control of appetite during food shortage.

Besides light, the most potent synchronizer of the master clock, feeding time also provides temporal cues to the circadian timing system, among others *via* secondary clocks in the brain. De Araujo et al. addressed this issue in nocturnal rats by investigating the phase-shifting effect of food access limited either to the daytime (resting period) or nighttime (active period) on clock gene expression in three hypothalamic regions, the suprachiasmatic nuclei (master clock), and the arcuate and paraventricular hypothalamic nuclei, two structures involved in the homeostatic regulation of food intake. The phase-shifting effects of time-restricted feeding on clock gene expression differ according to the degree of food restriction and the hypothalamic region considered, the arcuate and suprachiasmatic nuclei being the most and least affected, respectively.

Daily torpor is an adaptive strategy to cope with low ambient temperature and food scarcity. In Djungarian hamsters, timing of daily torpor is controlled by the master clock in the suprachiasmatic nuclei. In their study, Cubuk et al. performed a transcriptomic analysis of the hypothalamus to determine intracellular pathways involved in the entrance into torpor. The authors found that expression of genes coding for collagen and procoagulation factor (vwf) is upregulated during the early state of torpor, suggesting specific protective molecular mechanisms occurring during torpor entrance.

Circadian clocks in peripheral tissues likely play a crucial role in keeping metabolic health. As reviewed by Aoyama and Shibata, the circadian clocks in skeletal muscles modulate not only muscle mass, muscle strength, myofiber type, but also lipid and carbohydrate metabolism. Both time-restricted feeding and exercise proved to be potent synchronizers of the muscle clocks. In addition, the authors reviewed which bone functions are regulated by the bone clock and how timed feeding and exercise modulate bone physiology and rhythmicity.

Pancreatic islets contain circadian clocks in endocrine cells producing glucagon (α) or insulin (β), but their circadian functions are not fully understood yet. Petrenko et al. developed a methodology to separate α and β cells, and study their self-sustained oscillations for several days in primary cultures. For that purpose, they generated a triple *reporter ProGlucagon-Venus/Insulin2 promotor-Cherry/PER2:Luc* mouse line expressing specific fluorescent reporters for α and β cells, and a luciferase reporter for monitoring circadian oscillations. This innovative method will be critical to assess *in vitro* circadian properties of α and β pancreatic cells and their respective role in the regulation of glucose metabolism.

Liver function shows dramatic fluctuations across a daily cycle, alternating between synthesis of energy stores (glycogen, lipids) during the active/feeding period and their utilization during the resting/fasting period. Several transcriptional networks connect metabolic pathways to the hepatic clockwork. De Cosmo and Mazzoccoli focus their review on the retinoid X receptors (RXRs), transcription factors activated by polyunsaturated fatty acids. The authors detail the transcriptional role of RXRs and their close interplay with the circadian clock in the liver.

In addition to shifting the circadian clockwork in the liver and other peripheral clocks, daytime-restricted feeding leads to morphological changes in hepatocytes of nocturnal rats. In the liver of rats on a time-restricted feeding schedule, De Ita-Perez and Diaz-Munoz investigated the daily changes in β-catenin, a protein regulating both cell–cell adhesion and gene transcription. The results show that the subcellular distribution of β-catenin and its phosphorylated forms is modified according to the daily cycle and such distribution is markedly changed by time-restricted feeding.

Circadian disturbances associated with high-fat feeding in male mice include increased daytime feeding and phase-advanced liver clock. The study of Palmisano et al. revealed that female mice fed with high-fat diet do not show these circadian disturbances, unless mice are ovariectomized, thus demonstrating that ovarian hormones have a protecting effect against the circadian disturbances produced by high-fat feeding. Gender difference in circadian regulation is of important relevance not only for understanding basic mechanisms but also for appropriate personalized medicine.

In humans, taking into account the daily variations of metabolic, stress, and immune markers is critical for medical diagnostics to discriminate between time-of-day effects from altered homeostatic regulation. The same holds for clinical investigations in sport studies. Along this line, Pritchard et al. report the importance of controlling chronobiological parameters (e.g., time-of-day of sampling, lag between awaking time and sampling) in the assessment of salivary cortisol and secretory immunoglobulin as biomarkers of stress in athletes.

The final three articles deal with the pathophysiological implications of the circadian–metabolic interactions for metabolic health. The growing pandemic in obesity and type 2 diabetes calls for preventive procedures and efficient control. Besides genetic susceptibility, overconsumption of high-energy food and sedentary lifestyle, circadian aspects have to be taken into account, such as time of meals and type of food. In their article, Blancas-Velazquez et al. describe the impact of a high-fat diet associated or not with diet-induced obesity on feeding patterns. The authors also review which homeostatic and hedonic pathways known to influence feeding rhythms may mediate the circadian effects of high-fat feeding.

Most neurodegenerative diseases are associated with several circadian disturbances. Alzheimer’s disease is a multifactorial pathology, leading to increased oxidative stress, β-amyloid deposits, and neurofibrillary tangles. Alzheimer’s disease patients typically display marked alterations in sleep–wake cycle, body temperature, and hormonal rhythms. Schmitt et al. studied the effects of β-amyloid on circadian rhythmicity, ATP levels, and oxygen consumption in various cell cultures. They demonstrate that β-amyloid lengthens the circadian period in human fibroblasts, human glioma cells, as well as mouse primary neurons. Furthermore, β-amyloid decreases ATP levels and oxygen consumption, while increasing the oxidized state. Together, these findings suggest that β-amyloid deposits during Alzheimer’s disease may participate in circadian disturbances.

In addition to metabolic pathologies and neurodegenerative diseases, cancer may have profound effects on circadian rhythmicity, in particular, at the molecular level. The review by Padmanabhan and Billaud describes the reprogramming of the molecular clockwork during different cancers, such as hematopoietic malignancies. Thereafter, they discuss how deregulation of the oncogene *Myc* may interact with the clockwork and intracellular metabolic sensors (AMPK and SIRT1), suggesting that novel antitumor treatments could target these interactions.

To conclude, this research topic encompasses a wide array of functional interactions between circadian rhythms and metabolism at the molecular, cellular, tissue, and organism level. Chronotherapeutic approaches are expected to open new avenues to treat the numerous pathologies involving circadian disturbances.

## Author Contributions

All authors listed have made a substantial, direct, and intellectual contribution to the work and approved it for publication.

## Conflict of Interest Statement

The authors declare that the research was conducted in the absence of any commercial or financial relationships that could be construed as a potential conflict of interest.

